# Supplementary material for: Development and Differentiation of Epididymal Epithelial Cells in Korean Native Black Goat
Source: Animals (Basel). 2020 Jul 25;10(8):1273. doi: 10.3390/ani10081273 (PMC7459902; doi:10.3390/ani10081273)
Supplement: Supplementary file 1 [file animals-10-01273-s001.pdf]

Table S1. Primary antibodies.

Type	Host Species	Against	Catalog No.	Company	Application
Polyclonal	Chicken	B1-VATPase	.	Gift from Dr. Sylvie Breton (Center for System Biology, Harvard Medical School)	1:1000 [1]
Monoclonal	Rabbit	Keratin 5	RM-2106	Thermo Fisher Scientific (Waltham, MA, USA)	1:200 [13]
Monoclonal	Mouse	ZO1	33-9100	Thermo Fisher Scientific (Waltham, MA, USA)	1:200 [16]

Table S2. Secondary antibodies.

Host Species	Against	Conjugated to	Catalog No.	Company	Application
Donkey	Chicken IgG	CY3	703-166-155	Jackson ImmunoResearch Laboratories, Inc (West Grove, PA, USA)	1:800
Donkey	Rabbit IgG	FITC	711-096-152	Jackson ImmunoResearch Laboratories, Inc (West Grove, PA, USA)	1:150
Donkey	Mouse IgG	FITC	715-096-150	Jackson ImmunoResearch Laboratories, Inc (West Grove, PA, USA)	1:150
